# Creation of a theoretically rooted workbook to support implementers in the practice of knowledge translation

**DOI:** 10.1186/s43058-023-00480-w

**Published:** 2023-08-18

**Authors:** Christine Fahim, Melissa Courvoisier, Nadia Somani, Fatiah De Matas, Sharon E. Straus

**Affiliations:** 1 Knowledge Translation Program, Li Ka Shing Knowledge Institute, St. Michael’s Hospital, Unity Health Toronto, 30 Bond St., Toronto, ON M5B 1W8 Canada; 2 Grand Challenges Canada at the Sandra Rotman Centre, Unity Health Network, MaRS Centre, Toronto, ON M5G 1L7 Canada

**Keywords:** Implementation practice, Knowledge translation, Theories, models and frameworks, Intersectionality, Guide

## Abstract

**Background:**

Few training opportunities or resources for non-expert implementers focus on the “practice” as opposed to the “science” of knowledge translation (KT). As a guide for novice implementers, we present an open-access, fillable workbook combining KT theories, models, and frameworks (TMFs) that are commonly used to support the implementation of evidence-based practices. We describe the process of creating and operationalizing our workbook.

**Methods:**

Our team has supported more than 1000 KT projects and 300 teams globally to implement evidence-based interventions. Our stakeholders have consistently highlighted their need for guidance on how to operationalize various KT TMFs to support novice implementers in “practising” KT. In direct response to these requests, we created a pragmatic, fillable KT workbook. The workbook was designed by KT scientists and experts in the fields of adult education, graphic design, and usability and was piloted with novice implementers. It is rooted in an integrated KT approach and applies an intersectionality lens, which prompts implementers to consider user needs in the design of implementation efforts.

**Results:**

The workbook is framed according to the knowledge-to-action model and operationalizes each stage of the model using appropriate theories or frameworks. This approach removes guesswork in selecting appropriate TMFs to support implementation efforts. Implementers are prompted to complete fillable worksheets that are informed by the Theoretical Domains Framework, the Consolidated Framework for Implementation Research, the Behaviour Change Wheel, the Effective Practice and Organization of Care framework, Proctor’s operationalization framework, the Durlak and DuPre process indicators, and the Reach, Effectiveness, Adoption, Implementation and Maintenance (RE-AIM) framework. As they complete the worksheets, users are guided to apply theoretically rooted approaches in planning the implementation and evaluation of their evidence-based practice.

**Conclusions:**

This workbook aims to support non-expert implementers to use KT TMFs to select and operationalize implementation strategies to facilitate the implementation of evidence-based practices. It provides an accessible option for novice implementers who wish to use KT methods to guide their work.

**Supplementary Information:**

The online version contains supplementary material available at 10.1186/s43058-023-00480-w.

Contributions to the literature

- Recognizing the limited resources available to support non-expert implementers in using knowledge translation methods to implement evidence-based practices, we created an open-access, fillable workbook.
- The workbook is framed according to the knowledge-to-action model and operationalizes each stage of the model using appropriate theories or frameworks, thus removing some guesswork for novice implementers.
- The workbook is rooted in principles of integrated knowledge translation and applies an intersectionality lens, which prompts implementers to how user needs affect implementation efforts.

## Background

The period required for research evidence to be implemented in practice can be very long, with estimates ranging up to 28 years [1]. The use of knowledge translation (KT) methods to implement research evidence can reduce this lag [2, 3]. KT involves the dynamic and iterative process of conducting knowledge synthesis, dissemination and exchange, and the ethically sound application of knowledge, in partnership with knowledge users, to improve healthcare, services, policy, and systems [3–6].

Globally, there have been repeated calls for capacity building in KT science and practice to leverage KT methods and rapidly scale implementation efforts at the patient and health system levels [7–11]. In 2011, members of our team performed a thorough literature review but were unable to identify any studies of national strategies to build KT capacity [12]. In response, we developed a three-stream national capacity-building program, which consisted of training for graduate students and postdoctoral fellows (stream 1); training in the basic principles of the science and practice of KT for researchers (stream 2); and training in the practice of KT for knowledge users interested in applying KT approaches to improve knowledge, build skills and inform decision making (stream 3) [12]. Importantly, our capacity-building streams distinguished between the “science” and the “practice” of KT. While implementation science is the study of methods to promote the uptake of research findings (e.g., which implementation strategy is more effective in bringing about the desired change?) [13], implementation practice is the *use* of KT methods and implementation strategies to facilitate uptake of research findings in practice [14] (e.g., using opinion leaders to promote uptake of clinical practice guidelines) and policy. We recognize that the implementation science can be conducted alongside implementation practice, but is not essential to “doing KT” [15]. Finally, we acknowledge the potential overlaps between implementation practice and quality improvement yet recognize these as related yet distinct disciplines. Drawing upon the definitions provided by Koczwara et al., quality improvement initiatives aim to “improve the quality, safety, or value of healthcare,” while implementation practice aims to “facilitate the systematic uptake of evidence-based interventions into practice and policy” [16] to improve care and strengthen the health system. In this work, we focus on the latter, while recognizing the synergies between implementation practice, science, and quality improvement.

In our experience, stream 3 capacity building presents significant challenges. Healthcare providers, program managers, and decision- and policymakers desire to use KT methods to support their practice, yet limitations of time and a paucity of pragmatic capacity-building training options constrain their ability to build KT knowledge and skills. In response, we created a practice of KT workbook which presents a set of fillable worksheets modeled on commonly used KT models, theories, and frameworks.

In this manuscript, we describe the components of this workbook, how it was developed, and how it is intended for use.

## Methods

### The knowledge translation program: history and role

The KT Program is situated in the Li Ka Shing Knowledge Institute at St. Michael’s Hospital-Unity Health Toronto in Canada [17]. Since its inception, the KT Program has supported, through its consultation service, more than 1000 KT projects and 300 teams globally to implement evidence-based interventions. Additionally, we offer training courses in the practice of KT, taken by > 10,000 individuals from > 20 countries [18]. In the past decade, dozens of teams have commissioned the KT Program to support the implementation of evidence into practice and to build capacity in KT TMFs. Through these consultations and through our interactions with course participants, we identified a need for pragmatic resources that leverage KT TMFs in a manner that is accessible to novice implementers. Subsequently, in partnership with the teams seeking our KT consultation services, we designed and refined resources to meet this identified need.

### Our approach to conducting pragmatic KT and intended audience

Theories, models, and frameworks are the backbone of KT methodology. Briefly, a theory can be used to describe predictive and causal mechanisms of a behavior or phenomenon, a framework can be used to organize and explain the factors that influence implementation and outcomes, and a model specifies the steps in the process for translating research into practice [19].

We used the knowledge-to-action process model to guide the structure of our workbook. At the center of the knowledge to action model is the knowledge creation funnel which includes the processes of knowledge inquiry, knowledge syntheses, and the creation of knowledge tools and products (e.g., guidelines, decision-making tools) to support the dissemination of evidence-based practices. Surrounding the funnel is an action cycle, which includes eight “stages". Each of these “stages” provides guidance on how evidence can be adapted, implemented, evaluated, and sustained in practice [3, 20].

In our workbook, we focus on the action cycle, rather than the knowledge creation funnel. One of the requirements for participation in our stream 3 training is that participants arrive to the course with an evidence-based practice that they wish to implement in a specific setting. Thus, we aim to support those who are already aware of a clinical practice, guideline, or behavior that they want to implement in their setting, rather than those aiming to generate new knowledge or synthesize a body of evidence.

Using the Consolidated Framework for Implementation Research typology [21], our intended audience includes leaders, implementation leads/team members, and/or innovation deliverers seeking to implement or evaluate an evidence-based practice (e.g., a clinician-scientist seeking to implement a clinical practice guideline in their organization, a policymaker seeking to implement a policy across regional hospitals). These individuals could be situated in an organization’s inner or outer setting, and implementers should use an integrated KT approach to identify and engage with the various stakeholders who will be impacted by the implementation (including innovation deliverers and recipients) to co-design, implement, and evaluate the evidence-based practice [22, 23]. Depending on the setting and context, these stakeholders could include, but are not limited to: patients, public, families and caregivers, healthcare providers, managers, opinion leaders, and policymakers. In particular, our workbook aims to support implementers who wish to use KT methodology but who have limited knowledge or expertise in its application. This workbook can also serve as a guide to trainees or graduate students who are interested in advancing their knowledge of KT methodology. Finally, this workbook was created using a health services research lens and the examples provided reflect clinical practice. Thus, those in healthcare or health services might find this workbook most useful, though the principles outlined in the workbook can be applied to practitioners in any field aiming to implement evidence-based practices.

### Selection of TMF to inform our workbook

Our workbook is rooted in the knowledge to action process model, though we recognize there are multiple implementation models that can alternatively be used to guide implementation practice [6, 19]. We opted to use the knowledge to action model, as it spans the entire implementation spectrum (i.e., includes knowledge creation, dissemination, adaptation, implementation, evaluation, and sustainability [6, 24]) and has been widely adopted as a guiding model by national and international health agencies and was based on a review of theories of planned action. In our Canadian context, the knowledge to action model is also recommended and used by the Canadian Institutes of Health Research (CIHR), Canada’s federal funding agency for health research.

Process models are used to describe or guide the process of moving research evidence into practice and are operationalized using theories, frameworks, or theoretical frameworks [19, 24]. There is often overlap in the content and use of theories, models, and frameworks (TMF) and a plethora of TMF to select from [19]. Esmail et al. identified 596 studies in their scoping review that reported the use of 159 KT TMFs. The majority of these TMFs (87%) were used in fewer than five studies, and 60% were each used in a single study [24]. It can therefore be considerably challenging to determine which TMF is suited for any given project or how to combine the use of multiple TMFs, particularly among those lacking familiarity with KT methods. Furthermore, there is little guidance for novice implementers or non-KT experts seeking to use TMFs to develop, implement, evaluate, or sustain evidence-based practices. Our learners suggested that having guidance on which TMF to use, and how to combine the use of multiple TMF, would be beneficial in supporting KT practice efforts [18].

**Fig. 1:**
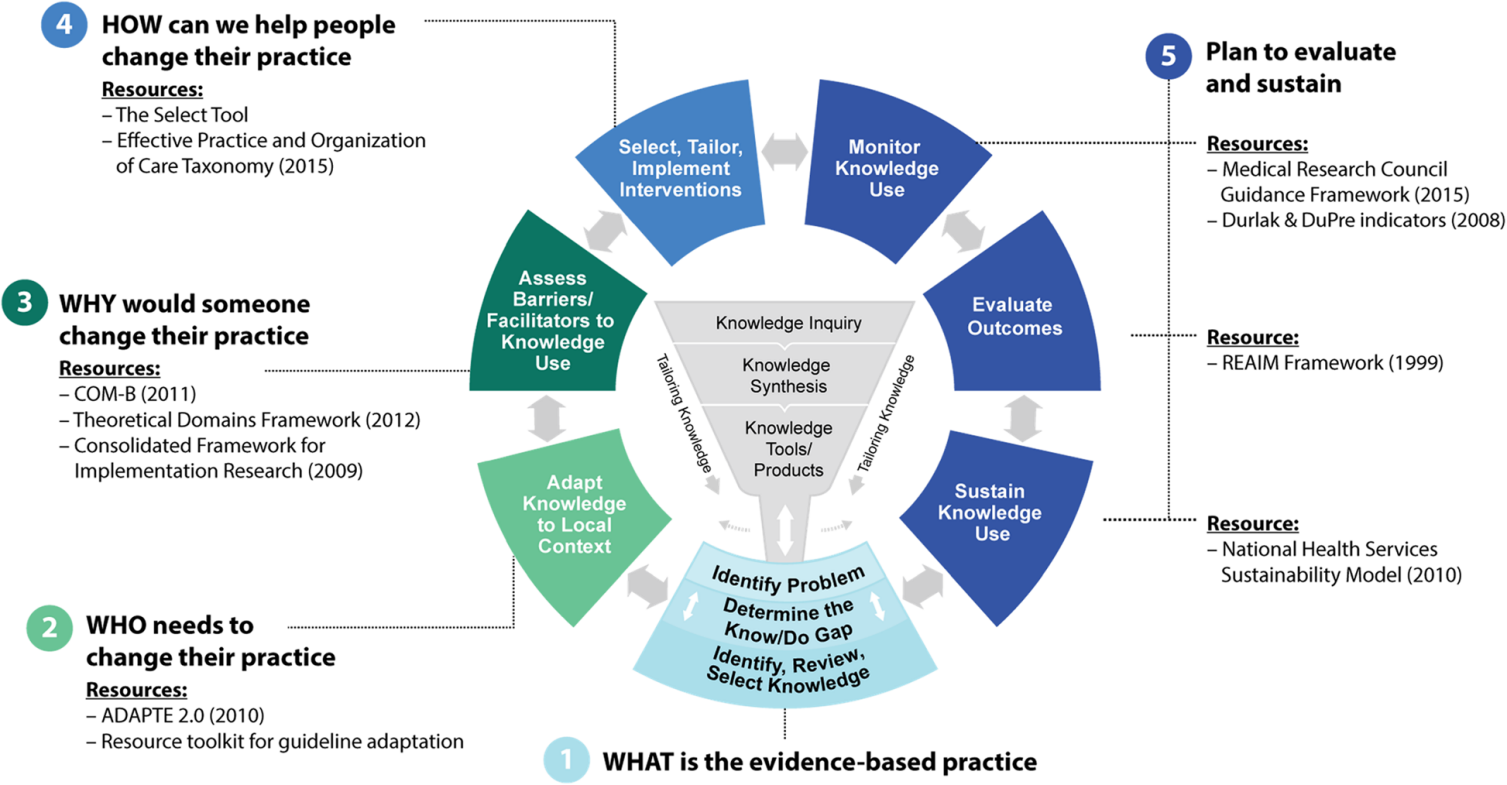
Operationalizing the knowledge to action cycle

In our courses, we operationalize each of the stages of the knowledge to action cycle with frameworks or theories that are commonly used, accessible, and, where possible, have tools and guidance to support their use (e.g., RE-AIM) [25–27]. For instance, one of the stages in the knowledge to action model is the *adaptation of evidence into the local context*, which can be operationalized using the ADAPTE process. The ADAPTE process provides users with a systematic approach to adapt evidence and is facilitated by a toolkit that can be used to ensure a guideline is appropriate to a particular context or setting [28]. Practitioners who perceive existing guidelines to be incompatible with their context or population can choose to use the ADAPTE toolkit to determine how to adapt the evidence to suit their needs. In Fig. 1, we depict our operationalization of the knowledge to action model.

### Simplifying the approach: creation of the workbook

Our course participants perceived Fig. 1 to be a useful starting point on how to utilize multiple TMFs to guide implementation, yet still found the process to be too complex to facilitate pragmatic use. In response, we aimed to further simplify the approach. Instead of referring to multiple knowledge to action “stages", we created a workbook that outlines five simplified steps to guide implementation.

- Step 1: WHAT is the evidence-based practice?
- Step 2: WHO needs to change their practice?
- Step 3: WHY would someone change their practice (or not)?
- Step 4: HOW can we help people change their practice?
- Step 5: PLAN for evaluation and sustainability.

Each of these steps has fillable worksheets, rooted in common TMFs and KT methods. Implementers are provided prompts to think through each of these steps when planning their implementation approach, considering the specific needs of their knowledge users and context.

### Usability testing

The workbook was usability tested with novice implementers who were recruited using a convenience approach via our networks [29]. Consenting implementers participated in a 1-h interview with a research coordinator. Using a “think aloud” interview process, implementers were asked to provide feedback on the content, format, layout, and clarity of the workbook. Interview data were recorded and reviewed by a research team member who extracted field notes based on usability feedback. Field notes were analyzed by two research staff using qualitative content analysis, guided by the framework approach [30]. Any coding discrepancies between − 1 and 0.6 (kappa) were discussed and resolved through a consensus meeting; subsequent transcripts were single-coded. Analyses were conducted using the Nvivo 11 software. Minor suggestions (e.g., add sub-headings to improve clarity) and major suggestions (e.g., add examples to illustrate each of the steps) agreed upon by at least 25% of the sample were incorporated. Participant feedback was iteratively incorporated into the workbook, and subsequent versions were circulated to elicit additional feedback.

## Results

### Results of usability testing

A total of nine participants usability tested the workbook. Participants were hospital (*n* = 4), government (*n* = 2), not-for-profit (*n* = 1), and research (*n* = 1) employees. Six participants worked at their organization for < 5 years, 2 for 5–10 years, and 1 for > 15 years. The majority of participants were from Ontario, Canada (*n* = 8), and one was from an international organization (*n* = 1). Participants included clinician-scientists and researchers who were novices to KT methodology; all participants were involved in at least one project that aimed to use KT methods to design or implement an evidence-based intervention. A total of 7 and 2 participants were women and men, respectively. Usability feedback is summarized in Appendix 1.

Usability testing results suggested the importance of including an example to guide implementers through the workbook prompts. To illustrate each of the five steps in the KT workbook, we provide an example from our Mobilization Of Vulnerable Elders (MOVE) study, which aimed to promote early mobilization of older adults in hospitals to reduce functional decline and adverse events [31]. A detailed example of how MOVES was designed and implemented, guided by the five steps of the workbook, is provided in Appendix 2.

### PKT workbook

#### Overarching principles guiding the workbook

**Fig. 2:**
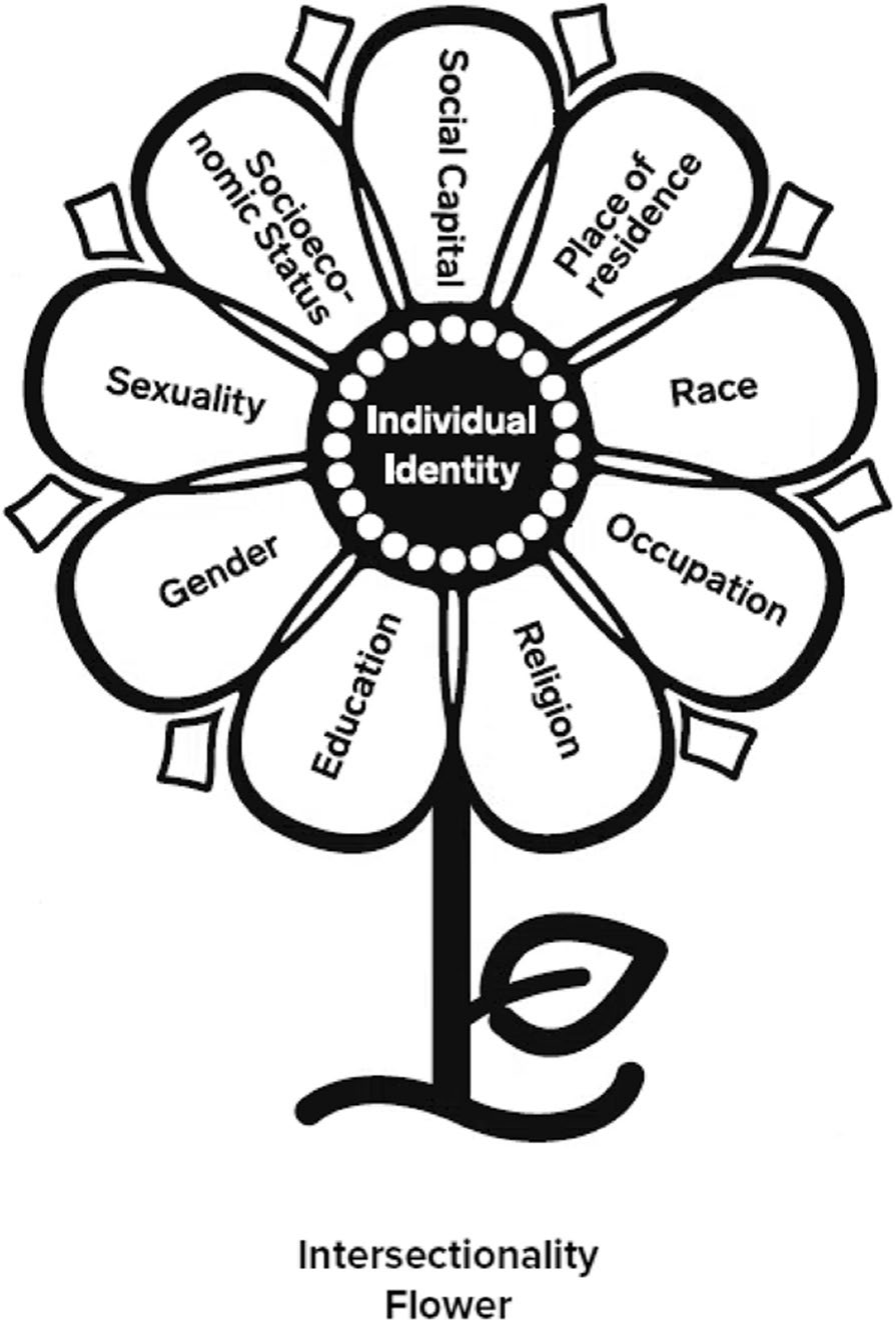
Intersectionality flower

Two overarching principles are used to guide each of the five steps of the workbook. First is the principle of using integrated KT, which emphasizes the need to involve knowledge users in the design, implementation, and evaluation of KT programs to optimize their potential impact [22, 23, 32]. Second is the use of an intersectionality lens to develop, implement, and evaluate interventions [33–36]. Intersectionality underscores the recognition that human experiences are shaped by a combination of social categories (e.g., gender, race/ethnicity, religion, occupation) that intersect and interact within existing power structures (e.g., political systems) (Fig. 2) [35, 36]. These concepts are further described on page 5 of the workbook.

##### Step 1: WHAT is the evidence-based practice?

In our experience, defining *what* needs to be implemented presents one of the biggest challenges to practitioners. Many practitioners are tasked with achieving a particular goal (e.g., improve outcomes for a certain patient population) rather than implementation of a specific evidence-based practice (e.g., improve handwashing practice among medical students). Successful implementation requires practitioners to specify in detail the individual evidence-based practices that need to be implemented. In our workbook, we prompt users to specify these practices and to outline the corresponding evidence supporting each practice. Throughout, we emphasize the importance of implementing evidence-based practice, or proceeding with caution when limited evidence exists. For each practice, users are prompted to specify the goals and targets that will be used to determine whether the implementation has been successful (e.g., within 6 months, achieve a handwashing compliance of 90% among medical students on the general internal medicine ward).

##### Step 2: WHO needs to change their practice?

Once implementers have clearly identified their “WHAT” (i.e., the evidence based practice), they are prompted to consider *who* needs to change their practice and what they need to do. Implementers are prompted to consider the various actors involved in an implementation process, which can include, but are not limited to: patients, caregivers, families, clinicians, and decision-makers at an organizational or policy level. Each actor group likely has different behaviors that need to change in order to facilitate change (for instance, physicians might need to increase referrals to specialized clinics to support patients with heart failure; patients need to attend these visits and be compliant with their treatment plans [37]). Implementers are also encouraged to form an implementation team, composed of 3–5 individuals who will support the day-to-day implementation process [38].

##### Step 3: WHY would someone change their practice (or not)?

Building on the exercise in step 2 that required implementers to identify all of the actors involved in implementation, step 3 prompts implementers to consider the barriers or facilitators to change for each of these actor groups. For instance, why would physicians change, or not change, their referral patterns, screening behaviors, or clinical assessment approaches? Theoretical frameworks can be used to assess the barriers and facilitators to change systematically, making it less likely for implementers to overlook a factor that might impede or facilitate implementation. Step 3 of the workbook is rooted in two commonly used theoretical frameworks, the Theoretical Domains Framework (TDF) and the Consolidated Framework for Implementation Research (CFIR).

The TDF and CFIR are both meta-frameworks, meaning they synthesize multiple theoretical constructs to present a set of factors impacting implementation. The TDF is synthesized from 128 theoretical constructs spanning 33 theories [25], while the CFIR is synthesized from 19 theories and leverages a systematic review of over 500 sources describing determinants of implementation [21, 39, 40]. The TDF and the CFIR were selected for use in our courses by an expert and experienced panel of KT scientists and practitioners; the panel opted to select the TDF to guide inquiry into the individual factors that influence behavior change, while the CFIR was selected to guide inquiry into the organizational and contextual factors that impact implementation of an evidence based practice.

Recently, our team led a research study that further confirmed the favourability and appropriateness of these two frameworks [36, 41]. We formed an interdisciplinary committee of KT, intersectionality, and clinical experts and prompted them to select theories and frameworks that they perceived as useful to operationalize each step of the knowledge to action cycle. Through a modified Delphi approach, the committee selected the Theoretical Domains Framework and the Consolidated Framework for Implementation Research as the preferred frameworks for operationalizing the implementation stages of the knowledge to action cycle as they are commonly cited, relatively easy to use and have tools to support their use [42].

In step 3, implementers complete a worksheet to describe the perceived barriers and facilitators for each evidence-based practice, considering the actors involved in each practice and recognizing that barriers and facilitators may be different for each group.

##### Step 4: HOW can we help people change their practice?

Implementation strategies are techniques that can be used to enhance the adoption of a practice. In the KT literature, they are also referred to as KT interventions or behavior change techniques [43–48]. Selection of appropriate strategies to facilitate implementation is an important, yet complex, process. Strategies should be linked to theory and evidence in order to address barriers and leverage facilitators [45]. Investing time in the selection of theoretically rooted, evidence-based strategies can reduce wasted resources and enhance the likelihood of achieving the intended behavior change. For instance, using reminders to promote handwashing on a hospital ward will not be an effective strategy, if the barrier to handwashing is lack of convenient access to handwashing stations.

The KT literature contains limited pragmatic guidance on how to link implementation strategies (the HOW) to overcome identified barriers and leverage facilitators [18, 43, 46, 47]. In response to this gap, we developed the SELECT tool [48] to support linkages between barriers to and facilitators of implementation strategies [43–46, 49].

The TDF can be supplemented with the COM-B, a commonly cited theory that suggests behaviors are impacted by motivation, opportunity, and capabilities [49]. The TDF factors can be categorized to fit these three behavioral components [25, 46, 49]. The COM-B, in conjunction with the TDF, can therefore be used to map identified barriers and facilitators to what is known as “intervention functions,” which put simply are the intervention categories that can be used to mitigate barriers and leverage facilitators. For instance, we can overlay the TDF to the COM-B to determine that use of *education* can be used to address barriers related to *knowledge*. However, how do we operationalize this *education* intervention?

The SELECT tool uniquely facilitates the next step by providing guidance on the specific implementation strategies that correspond to an intervention function [50]. In the workbook, implementers are guided to match implementation strategies (HOW) with their identified barriers and facilitators (WHY).

First, implementers are guided to “check” all of the TDF factors identified in step 3 as a barrier or facilitator, respectively, to behavior change for an actor group. Next to each TDF domain, we listed all of the corresponding intervention functions on the COM-B that correspond to that domain. Implementers are prompted to count the number of times an intervention function corresponds to the “checked” TDF domains in order to determine which intervention functions to prioritize. Once the intervention functions have been identified, implementers select the specific implementation strategies that are appropriate for their actors and context [50]. For instance, implementers who are prompted to select an *education* intervention function can select from the strategies of conducting educational meetings or distributing educational materials. Implementers who are prompted to select a *training* intervention function can select from the strategies of working with educational institutions to train providers, using train-the-trainer strategies, conducting educational outreach visits, conducting training using modeling to show how to perform an ideal practice, or providing clinical supervision.

In the Appendix of the workbook, we provide a detailed example of how to complete the SELECT tool to facilitate the mapping process between TDF-identified WHY domains, and the HOW. In addition, we include a worksheet rooted in the CFIR that guides implementers to identify contextual and systems-level barriers to and facilitators of implementation [40]. Implementers are encouraged to identify barriers and facilitators using the CFIR and map them to corresponding strategies using the Expert Recommendations for Implementing Change (ERIC) database [47].

Once the implementation strategies have been selected, implementers are guided to operationalize each one. The operationalization exercise is rooted in guidance from Proctor et al. on how to specify and report implementation strategies [51]. Implementers are guided to consider who needs to do what, how often this needs to happen (i.e., temporality), how much needs to be given (i.e., dose), and how the strategy can be implemented in a high-quality manner (e.g., whether instructors first need to be trained or whether auditors need to be given access to electronic records to complete audit and feedback reports).

##### Step 5: PLAN for evaluation and sustainability

As the final step of the workbook, implementers are encouraged to consider planning for evaluation and sustainability. With respect to evaluation, implementers are prompted to assess the implementation quality (i.e., how well their implementation strategies were implemented). Assessing implementation quality can provide initial “signals” of whether implementation is going according to plan. By assessing implementation quality early and regularly, we can make modifications to our implementation strategies to improve the likelihood of achieving our desired behavior change, and subsequently, our desired outcomes. A table in the workbook provides suggestions for implementation quality outcomes (rooted in the Durlak and DuPre process evaluation framework) that can be used to ensure the implementation is on track before clinical or systems outcomes are evaluated [52, 53]. Finally, implementers are encouraged to plan for sustainability upfront and are presented with a table that outlines sustainability factors that can be considered during implementation planning.

## Discussion

We have presented our approach to building pragmatic KT capacity among teams seeking to implement evidence-based interventions. There are many strengths to this approach, the primary one being an emphasis on embedding integrated KT in each step of the implementation process. Although the use of an integrated KT approach from project inception requires an investment of time and resources to build relationships and rapport with key knowledge users, it facilitates a more streamlined, efficient implementation process than would otherwise be possible [3]. Additionally, the use of intersectionality-enhanced TMF addresses a long-standing gap in the field of KT, which has largely neglected intersectionality and equity considerations when developing or implementing KT interventions [36, 42, 54–56].

This approach is well aligned with others that have been presented in the implementation science literature. In 2017, we supported Health Canada (the federal government department responsible for the health of Canadians) to create a Knowledge Translation Planner, which describes the use of knowledge-to-action to plan for knowledge dissemination and implementation [57]. Similarly, French et al. described a four-step approach to developing KT interventions [58]. Curran et al. created a teaching tool to explain implementation science terminology using plain language (e.g., implementation science helps us to “do the thing”; implementation outcomes describe “how much” and “how well” we “do the thing”) [59]. Our KT primer is unique in presenting a workbook that implementers can complete as they work through these implementation steps. As such, this tool takes the guesswork out of selecting and operationalizing commonly used TMFs and represents an accessible option for novice implementers aiming to use KT methods to guide their work. We are now converting the workbook into an online tool to further support its uptake and use. Implementers who intend to use this workbook can consider conducting an evaluation to determine whether use of the workbook impacted changes in attitudes, knowledge, and skills related to KT methods or use of theory-rooted implementation strategies [11].

There are some limitations to the approach outlined here. First, the workbook does not include all of the various TMFs that can be used to guide implementation efforts; rather, we selected TMFs that are widely cited in the implementation literature, which would provide numerous examples to implementers. Second, additional efforts are needed to provide guidance on how to adapt knowledge to the specific context (stage 2 in the knowledge-to-action cycle). Finally, the SELECT tool was created through a prioritization exercise with implementation experts. KT experts have recently proposed discrete choice experiments and other methods to strengthen the prioritization methods used in determining the most salient barriers and facilitators (and corresponding implementation strategies) that are likely to elicit the desired change [59–62]. However, such methods require significant time and resources that may not be available to all implementers; the COVID-19 pandemic has further highlighted the need to find methods efficiencies to accelerate implementation [63]. The SELECT tool could be strengthened by incorporating these approaches to guide implementers in prioritizing identified strategies for implementation.

## Conclusions

We have presented a pragmatic KT workbook that can be used to support implementers in their practice of KT through commonly cited implementation TMFs, with consideration of an integrated KT and an intersectionality lens. We anticipate that this workbook will improve the accessibility of KT methods to novice implementers or non-KT experts and will thus support the transfer of evidence into practice.

### Supplementary Information

**Additional file 1.**

**Additional file 2.**

**Additional file 3.**

## Data Availability

Not applicable.
