# Supplementary material for: Creation of a theoretically rooted workbook to support implementers in the practice of knowledge translation
Source: Implement Sci Commun. 2023 Aug 18;4:99. doi: 10.1186/s43058-023-00480-w (PMC10436469; doi:10.1186/s43058-023-00480-w)
Supplement: Supplementary file 1 — Additional file 1. [file 43058_2023_480_MOESM1_ESM.docx]

**Appendix A: Usability Feedback**

We present below the major feedback suggested by at least n=3 participants, all of which were integrated into the workbook. Minor feedback suggested by fewer than 3 participants (e.g., rewording, providing a definition) was also incorporated, as feasible.

| Category | Overall recommendation for improvement | Specific change suggested | Number of people (Participant IDs) |
| --- | --- | --- | --- |
| Content | Participants noted that a more detailed introduction would be helpful | KTA cycle should be included in workbook to indicate where each activity links to KTA cycle | 3 (P2, P4, P6) |
|  | Participants noted that the workbook could provide more instructions and guidance for novice KT implementers | Could have pages of instructions at beginning of workbook to explain how to use workbook for new KT implementers. Need simpler guidance: how to use it? Who should use it? Should you use it as a group? When should it be used? Are there other considerations? | 3 (P3, P4, P8) |
|  |  | Include more detail about iKT. | 3 (P1, P5, P6) |
|  | Participants noted that it would be helpful to link the (research) project cycle to the workbook steps | Have flow chart or Gantt chart that would indicate trajectory of research project and identify which step of workbook could be used for each stage of research project. Indicate approximately how long each step would take. | 3 (P3, P4, P8) |
|  | Participants noted that more examples would be helpful | Include examples of different types of projects (eg. by intervention like medication vs. resource or by project category like health services, public health, policy). | 4 (P2, P3, P4, P5) |
|  | Participants suggested adding recommended readings and other additional resources for novice implementers to reference | - | 3 (P2, P4, P6) |
|  | Participants suggested simplifying the language to make it easier to understand (for non-academics) | - | 4 (P4, P5, P6, P7) |
