# Supplementary material for: Creation of a theoretically rooted workbook to support implementers in the practice of knowledge translation
Source: Implement Sci Commun. 2023 Aug 18;4:99. doi: 10.1186/s43058-023-00480-w (PMC10436469; doi:10.1186/s43058-023-00480-w)
Supplement: Supplementary file 2 — Additional file 2. [file 43058_2023_480_MOESM2_ESM.docx]

**Appendix B: Steps in the Implementation Workbook**

| **Step of workbook** | **Example from Mobilization Of Vulnerable Elders (MOVES) project** | **Stage of knowledge-to-action model and use of integrated KT** |
| --- | --- | --- |
| **STEP 1: WHAT is the evidence-based practice** | | |
| What is the gap to be addressed? | Older adults admitted to hospital are not being assessed for mobility or mobilized to reduce functional decline | Identify problem  Integrated KT |
| What is the evidence-based practice that you will use to address this gap? | 1. Assess mobility within 24 hours of hospital admission 2. Mobilize patients at least 3×/day 3. Use progressive, scaled mobilization tailored to each patient’s ability | Determine the know/do gap  Identify, review, select knowledge |
| What is your goal? | Process outcome:  >10% increase in proportion of patients with early mobilization  Clinical/patient outcomes:  Improved patient satisfaction  Decreased length of stay in hospital  Decreased functional decline | [Plan to] evaluate outcomes  Integrated KT |
| **STEP 2: WHO needs to change their practice** | | |
| Who needs to change in order for the evidence-based practice to be implemented? | Providers: need to change practice to align with MOVE program  Patients: need to agree to mobilization  Policy-makers/managers: need to agree to provide resources/space for mobilization  Caregivers: need to support patients in early mobilization | Determine the know/do gap  Adapt knowledge to local context |
| Who will benefit from this change in practice? | Patients will benefit from early mobilization by experiencing reduced functional decline and fewer negative outcomes during hospital stay |  |
| Who is on the implementation team at your site? | We assembled a team of five individuals who led implementation: a patient representative, an implementation specialist, 2 geriatricians and a nurse practitioner | Integrated KT  Intersectionality lens |
| Who else is needed to support implementation? What supports are available for implementation? | Need buy-in from hospital management, resources to support implementation |  |
| Considering intersecting factors, are there other stakeholders involved in your implementation project? | Need to determine how intersecting factors may affect patients’ vulnerability and/or willingness to participate in mobilization |  |
| **STEP 3: WHY would someone change their practice (or not)** | | |
| Why are individuals likely to change, or not? | Example barrier from physician: “It’s not my job to mobilize patients”  Example barrier from patient: “I’m afraid I’ll fall if I move while I’m in hospital”  Example facilitator from physician: “Once I started encouraging patients to get up, many did it on their own” | Assess barriers to/facilitators of knowledge use  Integrated KT Intersectionality lens |
| **STEP 4: HOW can we help people change their practice** | | |
| How will you overcome identified barriers and leverage facilitators? | We will redefine staff roles and provide staff training  We will educate staff, patients and caregivers on the benefits of mobilization  We will conduct an audit of the proportion of patients with early mobilization and provide feedback to staff and managers | Select, tailor, implement interventions  Integrated KT  Intersectionality lens |
| How will you operationalize your implementation strategy? | One-on-one coaching was used to provide strategies for mobilization  Team huddles were used to reflect on audit and feedback concerning mobilization and to identify opportunities to optimize the approach | Select, tailor, implement interventions  Monitor knowledge use |
| **STEP 5: PLAN for evaluation and sustainability** | | |
| What implementation outcomes will you plan to assess? | We will evaluate:   - Dose: Did we deliver the number of training and education sessions that we aimed to deliver? - Adherence/fidelity/adaptation: Were the training, education, and audit and feedback strategies delivered as planned? If not, what was changed? - Quality of delivery/participant responsiveness: Were physicians and patients/caregivers satisfied with the training, education and feedback strategies? Why or why not?   Reach: Did we train/educate/provide feedback to the number of physicians and patients/caregivers that we aimed to reach? | Evaluate outcomes  Sustain knowledge use |
| What are the other process and clinical, system outcomes that you will use to determine success of implementation? | We will evaluate the following process outcomes:   - Rate of mobilization - Characteristics of participants mobilized (or not) - Characteristics of providers participating in mobilization (or not) - Characteristics of sites participating in mobilization (or not) - Patient, provider knowledge of why mobilization is important   We will evaluate the following patient and system outcomes:   - Length of acute care required - Length of hospital stay - Rate of discharge - Duration for delirium - Risk of depression - Return to independent functional status | Evaluate outcomes |
| How will you plan for sustainability? | We will:   - Assess policymakers’ priorities and will advocate for mobilization to continue to be regarded as a priority - Assess competing quality improvement initiatives at hospitals/organizations and aim to identify pathways to integrate strategies across initiatives to reduce burden on patients and providers - Assess and aim to optimize workflow to include mobilization as routine practice - Form a sustainability working group to monitor mobilization rates - Develop indicators to assess success of sustainability | Sustain knowledge use |
